# Adult physical, sexual, and emotional abuse and postpartum depression, a population based, prospective study of 53,065 women in the Norwegian Mother and Child Cohort Study

**DOI:** 10.1186/1471-2393-14-316

**Published:** 2014-09-08

**Authors:** Marie Flem Sørbø, Hilde Grimstad, Johan Håkon Bjørngaard, Mirjam Lukasse, Berit Schei

**Affiliations:** Department of Public Health and General Practice, Faculty of Medicine, Norwegian University of Science and Technology, (NTNU), Postbox 8905, N-7491 Trondheim, Norway; Forensic Department and Research Centre, St. Olavs University Hospital Trondheim, Brøset, Norway; Department of Health, Nutrition and Management, Faculty of Health Sciences, Oslo and Akershus University College of Applied Sciences, St. Olavs plass, Postbox 4, Alnabru, N-0130 Oslo Norway; Department of Gynecology, St. Olavs University Hospital, Trondheim, Postbox 3250, Sluppen, Trondheim, N-7006 Norway

**Keywords:** Adult Physical, Emotional, Sexual abuse, and recent abuse, Postpartum Depression (PPD), Edinburgh Postnatal Depression Scale (EDS), The Norwegian Mother and Child Cohort Study (MoBa)

## Abstract

**Background:**

Postpartum depression (PPD) has detrimental consequences to the women, their infants and families. The aim of the present study was to assess the association between adult abuse and PPD.

**Methods:**

This study was based on data from 53,065 pregnant women in the Norwegian Mother and Child Cohort Study (MoBa), conducted by the Norwegian Institute of Public Health. Women were recruited through a postal invitation in relation to a routine ultra-sound invitation at week 18 of gestation. Exposure to adult emotional, sexual, physical abuse was based on self-report at week 30, also differentiating if the perpetrator was known or a stranger, and whether the abuse was recent or not (<12 month since abuse). PPD was measured with a four items version of the Edinburgh Postnatal Depression Scale (EDS) at six months postpartum. The associations between different types of adult abuse and PPD were performed with logistic regression, adjusting for age, parity, civil status, education, child abuse, social support, and depression prior to pregnancy.

**Results:**

Altogether, 11% had PPD, and 19% had been exposed to adult abuse. Women reporting adult abuse had an 80% increased fully adjusted odds of PPD (OR 1.8 95% CI 1.7-1.9) compared to non-abused women. There was a tendency towards higher odds of PPD for women reporting combinations of adult abuse (emotional, sexual and physical), as compared with those reporting sexual, emotional or physical abuse only. Exposure from known perpetrator was more strongly associated with PPD than exposure from an unknown perpetrator. Compared with women without adult abuse, the fully adjusted odds of PPD was 2.6 (95% CI 2.4-2.9) higher for women with any recent adult abuse and 1.5 (95% CI 1.5-1.7) higher for women with any adult abuse, but not recent.

**Conclusions:**

The results from this large prospective population-based cohort study support initiatives aiming to assess and adequately address abuse when counseling and treating women of PPD.

**Electronic supplementary material:**

The online version of this article (doi:10.1186/1471-2393-14-316) contains supplementary material, which is available to authorized users.

## Background

Postpartum depression (PPD) affects around 13% of women giving birth, and occurs within one year of childbirth [1, 2]. PPD is a public health concern, for which the consequences to the woman and her infant have been well established with both short and long term effects [3]. PPD negatively influences the experiences of motherhood and breastfeeding [4], it may cause impaired bonding with the infant [5], and altered relationship with the partner [6, 7]. Furthermore, increased risk of future maternal depressions [8] and adverse psychiatric outcomes in adolescent offspring has been demonstrated [9].

Known risk factors for developing PPD include a history of abuse as a child or an adult and overall 35% of the women worldwide have experienced partner or non-partner abuse [10–12]. Several studies show that women reporting lifetime abuse, recent intimate partner abuse, or child abuse are considered more prone to develop PPD than their counterparts [10, 13–16]. The combination of abuse and depression is complicated, as both stressors put women at elevated risk for health problems and adverse pregnancy outcomes [7, 17]. Abuse of women and PPD can be prevented, thus increased knowledge facilitating prevention is important. The associations between abuse and PPD have mostly been studied in cross sectional designs [18, 19]. For example, in a recent review by Beydoun et al only two of seven studies were prospective [20]; similarly two out of six in Wu et al’s meta-analysis had a prospective design [21]. These studies included only small samples and did not take into account other well known risk factors for PPD, such as previous depression and child abuse. We wanted to prospectively explore these associations in a large population, also including several possible confounding factors. Our primary objective was to investigate the association between different types of adult abuse, emotional, sexual and physical, as singular or combined exposures, and PPD. Secondly, we wanted to explore whether the associations differed if the perpetrator was known or a stranger to the woman.

## Methods

### Study population

**Figure 1:**
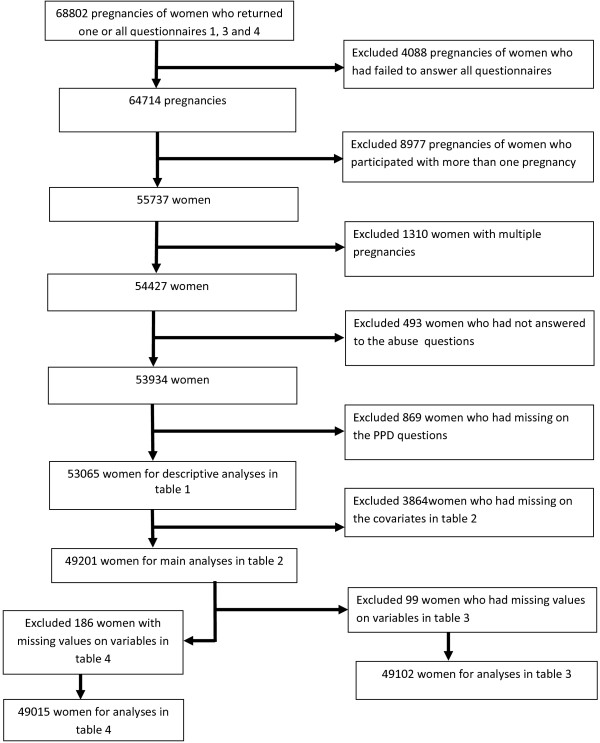
**Flow-chart of inclusion.**

Our study uses data from the Norwegian Mother and Child Cohort Study (MoBa), a prospective population-based pregnancy cohort study conducted by the Norwegian Institute of Public Health. The inclusion period was from 1999 to 2008. Hospitals with more than 100 births annually were invited to collaborate in the MoBa study and 70% of all pregnant women in Norway during this period were invited to participate. A total of 90,700 mothers and 108,000 children agreed to participate in the MoBa study. The response rate was 40.6%. All pregnant women in Norway are offered a routine ultrasound screening at week 18 of gestation at their local hospital [22]. Together with the ultrasound appointment, the women received a postal invitation that included an informed consent form, the first questionnaire and an information brochure. A detailed protocol of the study including the consent can be found elsewhere ( http://www.fhi.no/morogbarn). Women who agreed to participate received extensive self-administrated questionnaires by post, both during pregnancy and after birth. The MoBa sample has been described in more detail elsewhere [22, 23]. Data from the questionnaires are linked to the Medical Birth Registry of Norway, which is based on a standardized form completed by midwives shortly after delivery. The inclusion and exclusion of the study population are shown in Figure 1. Our study population consists of women who had filled in three questionnaires, at 18 and 30 of weeks of gestation, and six months postpartum. For women who participated more than once, information from their first pregnancy was included. Only women with singleton pregnancies and women who had answered a minimum of one of the abuse questions were included in the study. Those having missing on the PPD questions were excluded, leaving a total of 53,065 women whose characteristics are described in Table 1.

**Table 1:** **Characteristics of the study population in the Norwegian Mother and Child Cohort Study, 1999-2008**

	Any adult abuse			
	Yes (10267)		No (42798)	
	No.	%	No.	%
**PPD**				
No	8370	82	38979	91
Yes	1897	19	3819	9
*n	10267	100	42798	100
**Age in years**				
14-19	95	1	577	1
20-24	1145	11	5162	12
25-29	3325	32	16012	37
30-34	4323	42	17291	40
≥35	1378	13	3756	9
n	10266	100	42798	100
**Education**				
Primary (9yrs)	324	3	838	2
Secondary (12yrs)	3493	36	12114	29
Higher ≤ 4 yrs	5141	52	25852	63
Higher > 4yrs	893	9	2584	6
n	9851	100	41389	100
**Living with partner**				
Yes	9633	95	41656	98
No	564	6	923	2
n	10197	100	42579	100
**Parity**				
0	5066	49	22158	52
1+	5201	51	20640	48
n	10267	100	42798	100
**Any child abuse**				
no	7091	69	36264	85
yes	3176	31	6534	15
n	10267	100	42798	100
**Prior depression**				
no	8928	87	40795	95
yes	1339	13	2003	5
n	10267	100	42798	100
**Social support**				
no	368	4	1396	3
yes	9564	96	39882	97
n	9932	100	41278	100

*n vary according to numbers of missing values within the different variables. N = 53,065.

A total of 3,864 women were excluded due to missing covariate data, leaving 49,201 women for analyses of the association between different types of adult abuse and symptoms of PPD in Table 2. Of those exposed to adult abuse, 99 women did not report if they knew the perpetrator(s) or not, leaving 49,102 women for analyses in Table 3. Of the women exposed to adult abuse, 186 women did not respond to the questions of recent abuse (last 12 months) or not, leaving 49,015 women for analyses in Table 4. The current study is based on version 4 of the data files released for research in 2008 from the MoBa study. Written informed consent was obtained from each participant at recruitment. The study was approved by The Regional Committee for Medical Research Ethics in South-Eastern Norway. The research was performed in accordance with the Strobe guidelines [24]. An outline of the Strobe guidelines is added in Additional file 1.

**Table 2:** **Logistic regression analyses of the association between types of adult abuse and postpartum depression**

		Model 1		Model 2		Model 3	
		OR	95% CI	OR	95% CI	OR	95% CI
	n_PPD*						
**No adult abuse (ref)**	3471	1.0		1.0		1.0	
**Physical only**	75	1.7	(1.4-2.2)	1.5	(1.1-1.9)	1.4	(1.1-1.8)
**Sexual only**	149	2.0	(1.7-2.4)	1.7	(1.4-2.1)	1.6	(1.4-2.0)
**Emotional only**	888	2.1	(2.0-2.3)	1.8	(1.7-2.0)	1.7	(1.6-1.9)
**Emot.-physical**	229	2.8	(2.4-3.2)	2.2	(1.9-2.6)	2.0	(1.7-2.3)
**Physical-sexual**	33	2.2	(1.5-3.2)	1.8	(1.2-2.6)	1.7	(1.2-2.5)
**Emot.-sexual**	156	3.6	(3.0-4.4)	2.9	(2.4-3.5)	2.3	(1.9-2.8)
**Emot.-physic.-sex.**	195	3.4	(2.9-4.0)	2.7	(2.3-3.2)	2.2	(1.9-2.6)
**Any adult abuse**	1725	2.4	(2.2-2.5)	2.0	(1.8-2.1)	1.8	(1.7-1.9)

Model 1 adjusted for age and parity. Model 2 adjusted for age, parity, civil status, child abuse, education. Model 3 adjusted for age, parity, civil status, child abuse, education, social support, and prior depression. *Numbers of women in each abuse category reporting PPD. Analyzed for complete cases on all variables. N = 49,201.

**Table 3:** **Logistic regression analyses of the association between perpetrator status (known/unknown) and postpartum depression**

		Model 1		Model 2		Model 3	
Perpetrator + any adult abuse		OR	95% CI	OR	95% CI	OR	95% CI
	n_PPD*						
**No adult abuse (ref)**	3471	1.0		1.0		1.0	
**Known only**	1353	2.3	(2.1-2.5)	2.0	(1.8-2.1)	1.8	(1.7-1.9)
**Unknown only**	102	1.5	(1.2-1.9)	1.6	(1.3-2.0)	1.5	(1.2-1.9)
**Unknown and known**	254	3.7	(3.2-4.3)	2.3	(1.9-2.6)	2.0	(1.7-2.4)

Model 1 adjusted for age and parity. Model 2 adjusted for age, parity, civil status, education, and child abuse. Model 3 adjusted for age, parity, civil status, education, child abuse, social support, and prior depression. *Numbers of women reporting PPD within each category of perpetrator/abuse. Analyzed for complete cases only, N = 49,102.

**Table 4:** **Logistic regression analyses of the association between time (recent/not recent) of adult abuse and postpartum depression**

		Model 1		Model 2		Model 3	
		OR	95% CI	OR	95% CI	OR	95% CI
	n_PPD*						
**No adult abuse (ref)**	3471	1.0		1.0		1.0	
**Any adult abuse, but not recent**	1160	2.0	(1.9-2.2)	1.7	(1.6-1.9)	1.6	(1.5-1.7)
**Any adult recent abuse**	527	3.6	(3.2-4.0)	2.9	(2.6-3.2)	2.6	(2.4-2.9)

Model 1 adjusted for age and parity. Model 2 adjusted for age, parity, civil status, child abuse, education. Model 3 adjusted for age, parity, civil status, child abuse, education, social support, and prior depression. *Numbers of women in abuse categories reporting PPD. Analyzed for complete cases on all variables, N = 49,015.

### Variables

#### Assessment of PPD

**Figure 2:**
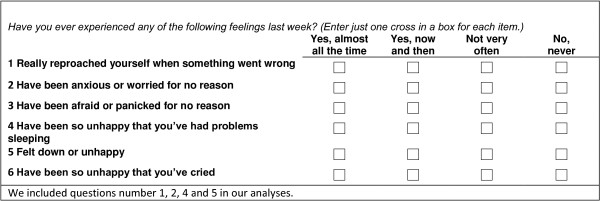
**Questions on Postpartum Depression in the Mother and Child Cohort Study.**

The Edinburgh Postnatal Depression Scale (EDS) is a self-rating scale designed to identify postpartum depression, and has two versions EDS-10 and EDS-5 [25]. The short-matrix 5 items version (EDS-5) has evidence of good psychometric properties, was primarily meant for research use, and has been translated into Norwegian and validated [26]. The questions on PPD were listed in questionnaire 4, and are displayed in Figure 2. In this paper we chose to use the four items identical to the items in the research version. The score ranges from 0 to 3 on each item, the latter indicating higher depression symptom score. We used a cut off score ≥ 6 which corresponded with a cut off at ≥ 10 in the EDS-10, and indicates a moderate level of PPD [27]. The PPD items 1, 2, 4 and 5 in Figure 2 were used in our analyses.

### Assessment of abuse

**Figure 3:**
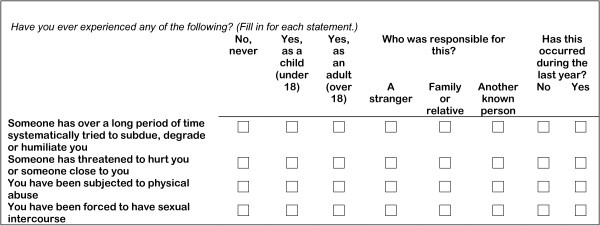
**Questions and response options on abuse and perpetrators in the Norwegian Mother and Child Cohort Study.**

The abuse questions and response options are shown in Figure 3. These questions were from the third MoBa questionnaire and responded to at approximately week 30 of gestation. The two questions of emotional abuse are almost identical to those in the Norvold Abuse Questionnaire [28], which measures mild and severe emotional abuse. The questions of emotional abuse in our study were merged into one variable. The question on sexual abuse and response options was based on a modified version of the sexual abuse question in the Abuse Assessment Screen (ASS) [29], a screening tool used in other Scandinavian studies [30, 31]. The question on physical abuse has been used in other studies, but is not validated [32, 33]. Women who answered yes to at least one of the adult abuse questions were defined as having suffered from any adult abuse. Likewise, women responding yes to one or more of the child abuse questions were defined as having suffered from any child abuse (used as a covariate for adjustment). Women could also indicate whether they had been abused the last 12 months or not (in our study categorised as recent abuse or not).

### Perpetrators

Women were given the opportunity to indicate if the abuse was committed by a stranger, a family member/relative or other known person (Figure 3). The two latter categories were merged into "known perpetrator". Furthermore, we included women reporting adult abuse from known perpetrator only, in the group; "known only". Those reporting abuse from stranger only were included in "stranger only" and, finally, women reporting abuse from both stranger and known perpetrators were included in the "stranger and known perpetrator" group.

### Possible confounding variables

Background information such as age, education, depression prior to pregnancy, social support, and civil status was collected from the first questionnaire at inclusion, in order to take into account possible confounding factors of the abuse-PPD association. Age was categorized into five groups (Table 1). Information about education was categorized into four groups: primary school (9 years), secondary school (12 years), two groups at college or university level, (≤4 years) or (>4 years). Women were asked to respond yes or no to whether, earlier in life, they had suffered from depression in a period of two weeks or more. Social support was defined as having anyone other than the partner the woman can ask for advice in a difficult situation, with three answering options; no, yes 1-2 persons, or yes, more than 2 persons, which we categorized into no or yes. Civil status was defined as living with partner or not. Information about parity was categorized into nulliparous, and women giving birth previous to this pregnancy (1+).

### Statistical analyses

Descriptive statistics of women exposed to adult abuse are presented in Table 1. Logistic regression analyses were used to estimate the associations between different types of adult abuse and PPD (Table 2), to estimate associations between perpetrators (known or unknown) of adult abuse and PPD (Table 3) and, finally, to estimate associations between time of abuse (recent or not) and symptoms of PPD (Table 4). We used three models adjusting for possible confounding factors. In Model 1, we adjusted for age and parity. In Model 2, we adjusted for age, parity, education, civil status, and any child abuse. Finally, in Model 3 all variables from Model 2 were included along with depression prior to pregnancy and experience of social support. The reference group for all analyses was women reporting no adult abuse. Adjusted odds ratios (OR’s) were presented for the different models with 95% confidence intervals (95% CIs) and analysed for complete cases only. The data programme PASW statistical 20 was used to conduct all analyses.

## Results

**Figure 4:**
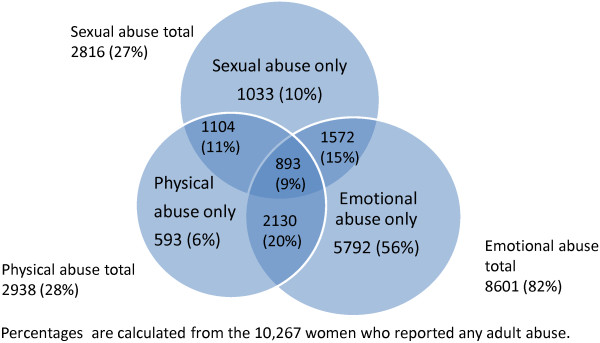
**Types of adult abuse and overlapping categories.**

Altogether, 11% of the women had PPD and 6% reported depression prior to pregnancy. Nineteen percent reported exposure to any adult abuse. Table 1 presents characteristics for the study participants by exposure to any adult abuse or not. Figure 4 shows the number of women reporting different types of adult abuse and combinations of adult abuse. Among the 2,938 women reporting adult physical abuse, 593 reported having suffered from adult physical abuse only. Of the 8,601 women reporting adult emotional abuse, 5,792 did not report having experienced any other abuse. While among those 2,816 reporting adult sexual abuse, 1,033 women reported adult sexual abuse only. Women reporting any adult abuse had an 80% increased fully adjusted odds of PPD symptoms (OR 1.8, 95% CI 1.7-1.9) compared to women without any adult abuse (Table 2). Women reporting combinations of emotional, physical and sexual abuse were more at risk of PPD than women reporting only one type of abuse. Women reporting three types of abuse; emotional, physical and sexual abuse, had a 120% increased fully adjusted odds of PPD (OR 2.2, 95% CI 1.9-2.6) compared to women reporting no adult abuse. Compared with women with no adult abuse, exposure from known perpetrator was more strongly associated with PPD than exposure from an unknown perpetrator (fully adjusted OR known perpetrator only 1.8, 95% CI 1.7-1.9 and unknown and known perpetrators OR 2.0, 95% CI 1.7-2.4 versus OR unknown perpetrator only 1.5, 95% CI 1.2-1.9) (Table 3). The odds ratio of PPD symptoms following any recent abuse in the fully adjusted model was 2.6 (95% CI 2.4-2.9) compared to no adult abuse, while those women reporting no recent abuse had an OR of 1.6 (95% CI 1.5-1.7) (Table 4). The associations between all types of adult abuse and PPD were attenuated when adjusted for confounding factors introduced in Models 2 and 3.

## Discussion

All types of adult abuse were strongly associated to PPD. Although the associations were attenuated with adjustment for possible confounding factors such as age, parity, child abuse, civil status, education, social support and depression prior to the pregnancy, the substantial associations remained in the fully adjusted models.

### Strengths and limitations

The prospective design of the study is a major strength. Also, the study included a large number of non- selected groups of pregnant women. The reporting of abuse and potential confounding factors were assessed during pregnancy hence reported prior to and unrelated to the reporting of PPD. We were also able to adjust for known risk factors of PPD, like child abuse, experience of social support, and previous depression, which is not always included in previous studies. There are also limitations. We were able to use only four out of five items in the validated EDS-5 version (research version) to measure PPD [27]. This may have influenced our estimated prevalence, but is probably less likely to have influenced our estimated associations. Also, diagnostic information of PPD would have been an advantage. However, given the prospective design of our study where potential confounding information was reported early in pregnancy and outcome assessment of PPD was reported after pregnancy, we believe that misclassification of abuse is not likely to be differential. The low response rate in the MoBa survey of 40.6% is a limitation. Nevertheless, a study investigating the possible effect of the low response rate on eight well-known exposure-outcome factors in the MoBa survey, concluded that prevalence estimates of exposures and outcomes were biased, but not estimates of associations between exposure and outcome [22].

### Comparing the results to other studies

#### Prevalence

Our results of PPD at 11% are in the range of the prevalence found in other studies in high income countries; including one Norwegian study using EPD-10 where 9% had PPD [34] and a meta-analysis where the prevalence of PPD was 13% [1].

### Dose-response association

Overall, women in our study exposed to more than one type of abuse had about a two to three fold increase in PPD, compared to non-abused women. Furthermore, the results indicate a dose-response association, as exposure to more than one type of abuse showed stronger associations to PPD than exposure to one type only. This is in agreement with other studies, where the strength of association increased with each additional type of violence experienced and with increased frequency of abusive acts [35, 36].

### Type, and timing of the abuse

Emotional abuse was the most commonly reported type of abuse in our study. Women exposed to emotional abuse only in our study had a slightly higher risk of PPD, compared with women exposed to either only sexual or only physical abuse. This is in accordance with other studies which indicate a higher risk of PPD among women exposed to emotional abuse compared to other types of abuse. For example, one clinical study of 200 women in Canada showed that emotional abuse but not physical or sexual abuse was found to be associated to PPD [18]. In our study the association between any abuse and PPD was stronger when the abuse was reported as recent compared to past experience. Our findings are consistent with previous studies linking recent abuse to PPD [14, 36, 37].

### Perpetrators

The literature is both scarce and inconclusive on the topic of perpetrators other than intimate partner. In our study women abused by known perpetrator only, or by known *and* stranger, were at higher risk of experiencing PPD than those abused by stranger only. This may be because exposure to abusive acts from a known person may have more detrimental effects to the women compared to abuse from a stranger. Reporting exposure of abuse from different perpetrators (both stranger and known) can imply strong association to PPD through different mechanisms. Being abused by a trusted person is likely to be more detrimental than being abused by an unknown person. Another possible explanation is that exposure to both known and stranger indicating more than one insult; hence contributing to the strong association. Recent studies indicate that recurrent acts of abuse are associated with an increased risk of PPD [36, 38], which correspond with our findings according exposure to abuse from different perpetrators. Nevertheless, results from three Canadian studies on PPD comparing abuse by partner and other perpetrators show diverse results [36, 39, 40]. In a population-based survey there was a strong association between abuse by partner and PPD, but no association was found of abusive acts perpetrated by other persons [36]. In contrast, one study showed no differences between women with and without PPD regarding who perpetrated the abuse (partner, other family member, or stranger) [39]. However, in the third study the odds of PPD were significantly greater among women abused by partner compared with those who did not experience partner abuse [40]. In the same study the perpetrators were equally to be partner or non-partner, and although not significant, abuse from other persons (family member, stranger or acquaintance), also showed a positive association to PPD.

## Conclusions

The women in our study commonly reported adult emotional, sexual, and physical abuse. All types were highly associated with PPD, either as singular types of abuse only, or in combination with other types. Furthermore, our findings showed that reporting abuse by a known perpetrator only, or by both a known and a stranger, showed stronger association with PPD compared to those abused from strangers only. Our findings highlight the importance of assessing and adequately addressing abuse when counseling and treating women with PPD.

## Electronic supplementary material

Additional file 1: **STROBE guidelines.** (PDF 99 KB)

## Abbreviations

(PPD): Postpartum depression
(MoBa): The Norwegian Mother and Child Cohort Study
(PPD): Postpartum depression
(EDS): The Edinburgh Postnatal Depression Scale
(ASS): Abuse assessment screen.
